# Assessing clinical relevance of off-target effects observed at suprapharmacological concentrations of SGLT2 inhibitors

**DOI:** 10.1172/JCI210991

**Published:** 2026-10-01

**Authors:** Simeon I. Taylor

**Affiliations:** Taylor Biomedical Consulting, North Bethesda, Maryland, USA.

**Keywords:** Clinical Research, Endocrinology, Metabolism, Diabetes, Intermediary metabolism, Pharmacology

**To the Editor:** Sodium-glucose cotransporter-2 inhibitors (SGLT2i) provide important clinical benefits in many disease states, including diabetes, chronic kidney disease, and heart failure. These clinical benefits must be balanced against treatment-associated adverse events. SGLT2i have been demonstrated to induce life-threatening ketoacidosis, especially in the setting of type 1 diabetes (1). The primary action of SGLT2i is to induce glucosuria. In response to the associated loss of calories in the urine, the body mobilizes stored calories by activating lipolysis in adipose tissue (2). Empagliflozin (25 mg for 4 weeks) increased mean fasting levels of β-hydroxybutyrate from 0.25 to 0.56 mmol/L in people with type 2 diabetes (3). In contrast, diabetic ketoacidosis is typically characterized by β-hydroxybutyrate levels in excess of 3 mmol/L (>15-fold higher than mean fasting levels of β-hydroxybutyrate in healthy individuals). This dramatic increase in ketone body biosynthesis is generally thought to be triggered because insulin deficiency disinhibits adipose tissue lipolysis followed by β-oxidation of fatty acids in the liver. The resulting increase in acetyl-CoA fuels an increase in ketone body synthesis (1, 3).

Abdualkader et al. (4) recently reported that empagliflozin binds directly to an allosteric site on 3-hydroxy-3-methylglutaryl-CoA synthase 2 (HMGCS2), thereby activating the enzyme. As HMGCS2 catalyzes one of the steps in the mitochondrial pathway of ketone body biosynthesis, Abdualkader et al. proposed that drug-induced allosteric activation of HMGCS2 may contribute to the pathogenesis of the SGLT2i-induced increase in ketogenesis. It is noteworthy that these experiments employed extremely high concentrations of empagliflozin, i.e., 100 μM for in vitro experiments with cultured human hepatocytes and 3,000 μM for in vitro drug affinity responsive target stability (DARTS) assays of ketogenic enzymes in liver lysates. To evaluate the clinical relevance of in vitro investigations of off-target drug effects, it is critical to compare the concentrations employed in the research with drug concentrations achieved in people treated with approved doses of the drug. According to the FDA-approved prescribing information, the *C_max_* for the 25 mg dose of empagliflozin is 687 nmol/L; plasma protein binding is 86.2%. Thus, the mean free concentration of empagliflozin in plasma is approximately 95 nmol/L at the time of *C_max_*. Thus, the empagliflozin concentrations used in the hepatocyte and DARTS experiments were > 1,000- and > 30,000-fold higher, respectively, than the calculated mean free plasma concentration at *C_max_* for people receiving the approved 25 mg dose. These calculations do not provide support for the clinical relevance of in vitro experiments conducted with concentrations of drugs so greatly exceeding free drug concentrations achieved in clinical practice.

The data reported by Abdualkader et al. (4) raise a number of additional issues: clinical data suggest that SGLT2i-induced glucosuria is a prompt response (5). In contrast, the drug-induced increase in ketone body levels is insignificant after 24 hours, whereas a 4-week course of empagliflozin induces a substantial increase in ketone body levels in both people with type 2 diabetes and in nondiabetic individuals (3). This raises questions about the clinical relevance of the report that increased levels of ketone bodies preceded drug-induced glucosuria (4).

The essential role of HMGCS2 in catalyzing ketone body biosynthesis implies that knockout of HMGCS2 would block the ability of empagliflozin to increase ketogenesis. However, this observation does not imply that activation of HMGCS2 mediates that increase.

Abdualkader et al. (4) provide evidence that empagliflozin increases HMGCS2 activity by approximately 20%–25%. That magnitude of activation would contribute at most an extremely small percentage of the > 1,000% activation of ketogenesis observed in ketoacidosis.

In vivo animal models, biochemistry, and structural biology are critical elements of translational research, including translational research to elucidate effects of drugs. Nevertheless, certain boundary conditions must be met to demonstrate relevance for human pharmacology. These boundary conditions are not met in studies of drugs at concentrations that greatly exceed those achieved in patients treated with the usual doses.

## Version 1 10/01/2026

Electronic publication
